# Ultrasound and 3D Skin Imaging: Methods to Evaluate Efficacy of Striae Distensae Treatment

**DOI:** 10.1155/2012/673706

**Published:** 2011-11-22

**Authors:** Mariella Bleve, Priscilla Capra, Franca Pavanetto, Paola Perugini

**Affiliations:** Department of Drug Sciences, School of Pharmacy, University of Pavia, Via Taramelli 12, 27100 Pavia, Italy

## Abstract

*Background*. Over time, the striae rubra develop into striae alba that appear white, flat, and depressed. It is very important to determine the optimum striae management. In order to evaluate the effectiveness of these therapies, objective measurement tools are necessary. *Objective*. The aim of this study is to evaluate if ultrasonography and PRIMOS can be used to obtain an objective assessment of stretch marks type and stage; furthermore, we aim to apply these techniques to evaluate the efficacy of a topical treatment. *Methods*. 20 volunteers were enrolled with a two-month study. A marketed cosmetic product was used as the active over one body area. The controlateral area with stretch marks was treated with a “placebo” formulation without active, as a control. The instrumental evaluation was carried out at the beginning of the trial (baseline values or *t*_0_), after 1 month (*t*_1_), and at the end of the study (*t*_2_). *Results*. PRIMOS was able to measure and document striae distensae maturation; furthermore, ultrasound imaging permitted to visualize and diagnose the striae. Statistical analysis of skin roughness demonstrated a statistically significant reduction of Rp value only in a treated group. In fact, the Rp value represented a maximum peak height in the area selected. These results demonstrated that after two months of treatment only the striae rubra can be treated successfully. *Conclusions*. This work demonstrated that the 22MHz ultrasound can diagnose stretch marks; PRIMOS device can detect and measure striae distensae type and maturation. Furthermore, the high-frequency ultrasound and the 3D image device, described in this work, can be successfully employed in order to evaluate the efficacy of a topical treatment.

## 1. Introduction

Striae Distensae or stretch marks (SDs) are a well-recognized, common skin condition that rarely causes some significant medical problems but is often a significant source of distress for those affected [1]. SDs arise from progressive or rapid stretching of the dermis (1) and appear along cleavage lines perpendicular to the direction of greatest tension in areas with the most adipose tissue [2, 3]. The classic anatomical sites affected include the abdomen and breasts for pregnancy-related striae, the outer thighs or lumbosacral regions in adolescent boys, and the buttocks, thighs, upper arms, and breasts in adolescent girls [2, 4]. Causes of SD are not clear, and a number of theories have been proposed.

Many authors had shown that mechanical effect of stretching is the main cause, leading to the rupture of the connective tissue framework (e.g., pregnancy, obesity, weight lifting) [5, 6]. Osman has denounced this theory, not finding any relationship between growth in abdominal girth in pregnant women and formation of SD [7]. Normal growth has been suggested as another cause, with these marks commonly developing during adolescence and associated with the rapid increase in size of particular regions of the body [8]. Similarly, obesity and rapid changes in weight have been shown to be associated with the development of SD. Moreover, Kogoj anticipated a damage of the tissues by a toxic way, resulting in striations [9]. Others suggested that SDs are a feature of high serum levels of steroid hormones induced from local or systemic steroid therapy or Cushing's disease. High steroid hormone levels have a catabolic effect on the activity of fibroblasts and decrease the deposition of collagen in the dermal matrix substance [10, 11]. SDs have been reported in patients with chronic liver disease, HIV, cachetic states, and anorexia nervosa [12–15].

Striae progress through three different stages of maturation: the acute stage is characterized by red and slightly raised striae (rubra), the subacute stage is characterized by purpuric striae, and the chronic stage is characterized by hypopigmented and atrophic striae (alba) [16]. In particular, striae rubra are flattened areas of skin with a pink-red hue that may be itchy and slightly raised. Then they are predisposed to increase in length and to acquire a darker purple color. Over time, the striae rubra develop into striae alba that appear white, flat, and depressed [17, 18]. Striae are separated from normal skin by a densely packed area of thin, eosinophilic collagen bundles, horizontal to the surface in a parallel orientation. Striae lack rete pegs, adnexal structures, and normal dermal undulations. In addition, hair follicles and other appendages are absent. Dermoscopy and histology studies have demonstrated that the color variations of striae are influenced by melanocyte mechanobiology and that striae formation occurs parallel to the wound-healing process of regular scar formation [16, 18]. Histologically, initial inflammatory changes are followed by a flattening and thinning of the epidermis due to underlying changes in the numbers and organization of collagen, fibrillin, and elastin fibers. Histological studies of mature striae reveal stretched collagen fibers aligned parallel to the skin surface, followed by subsequent loss of collagen and increased flattening of rete ridges. Reduced amounts of fibrillin surrounding the dermal-epidermal junction (DE), reduced elastin in the papillary dermis, and reorganization of elastin and fibrillin fibers in deep dermis contribute to the atrophied appearance of striae [17, 19].

In order to improve stretch marks quality, multiple therapies are under development. Treatment of striae varies and includes topical tretinoin, hydrant creams, topical oil massage, glycolic acid, and trichloroacetic acid [20–26]. Newer treatments involve lasers, including the 308 nm excimer laser, intensed pulsed light, and 585 nm flash-lamped laser [2, 17, 27–29].

It is important that these therapies are evaluated in clinical trials to determine the optimum SD management. In order to evaluate the effectiveness of these therapies, objective measurement tools are necessary.

Especially in clinical trials, objective evaluation is more reliable than subjective evaluation. In this direction, high-frequency ultrasound and surface roughness evaluation can be used together to the evaluation of SD improvement after treatment.

Initially ultrasonography was utilized to depict normal skin in a rapid and noninvasive way as cross-section images, especially for the measurement of skin thickness. Apart from the measurement of skin thickness, the main indications for ultrasonography in dermatology are the monitoring of the course and therapeutic efficacy of the disease treatment with skin sclerosis (e.g., morphea, systemic scleroderma, scleroderma like diseases) and the studies on the effects of topical and systemic drugs on the skin [30].

Currently, the evaluation of microrelief roughness and their modification is often only evaluated subjectively by means of a scale (Fitzpatrick skin type for striae distensae). But, several objective measurement tools for skin surface roughness can be distinguished. The majority of these tools use a silicone replica of the skin, after which the topography of the replica is measured. This indirect measurement is performed by use of mechanical, optical, laser, transparency or interference fringe projection profilometry [31–36]. The use of replicas is time-consuming and, therefore, not feasible in an outpatient setting. In addition, air bubbles, gravity, and patient movement may have an effect on the quality of the replica [37]. Another measurement method is the use of skin biopsy specimens but this technique is invasive [38]. In the last years, the use of a measuring device (PRIMOS) has permitted the *in vivo* evaluation of human skin surface topography that represents the three-dimensional (3D) organization of the dermis and the subcutaneous tissue. The surface topography can be considered a mirror of the functional status of the skin. In order to assess the success of an SD therapy the depth, variation and distribution of the furrows need to be measured prior and after treatment. Several articles have reported on the use of this instrument. First of all, the PRIMOS has been used in the cosmetic industry to measure human skin topography and volume of wrinkles. In addition, it has been used to objectively evaluate the depths of acne scars. Roques et al. successfully tested the PRIMOS in two patients with scars and recommended further testing of this assessment tool [39, 40].

The aim of this study is to evaluate if ultrasonography and PRIMOS can be used to obtain an objective assessment of stretch marks type and stage; furthermore, we aim to apply these techniques to evaluate the efficacy of topical treatment.

## 2. Materials and Methods

### 2.1. *In Vivo* Study Design

The study has been carried out according to Helsinki declaration (Ethical Principles for Medical Research Involving Human Subjects). Twenty healthy volunteers, of both sex, between 22 and 38 years old, showing stretch marks on buttocks, lateral abdomen, breast, and internal and external thights were selected according to the following inclusion criteria: people who have striae rubra and/or striae alba, good general health; absence of cutaneous diseases; and people who do not show pigmentary lesions or other lesions on the interest area that could interfere with the evaluation study.

Volunteers were enrolled with a two-month study. A marketed cosmetic product was used as the active which was applied once daily by massage over one body area. The controlateral area with stretch marks was treated with a “placebo” formulation without active, as a control. The instrumental evaluation was carried out at the beginning of the trial (baseline values or *t*_0_), after 1 month (*t*_1_), and at the end of the study (*t*_2_).

All measurements were made in an air-conditioned room with controlled temperature and humidity (T 22°C, r.h. 50 ± 5%); subjects were preconditioned for at least 15 minutes before the measurements.

### 2.2. High-Frequency Ultrasound Scanning

The high-frequency scanners available today operate at frequencies between 20 MHz and 1-2 GHz. The optimal frequency range for dermatological questions is between 20 and 100 MHz [41].

Using a 20 MHz transducer it is possible to visualize structures up to 6-7 mm in depth. This means that the zones of diagnostic interest are covered, that is, epidermis, corium, and one portion of subcutaneous fatty tissue. Particularly, if the subcutis is not very well developed, evaluation of the muscle fasciae is also possible.

**Figure 1:**
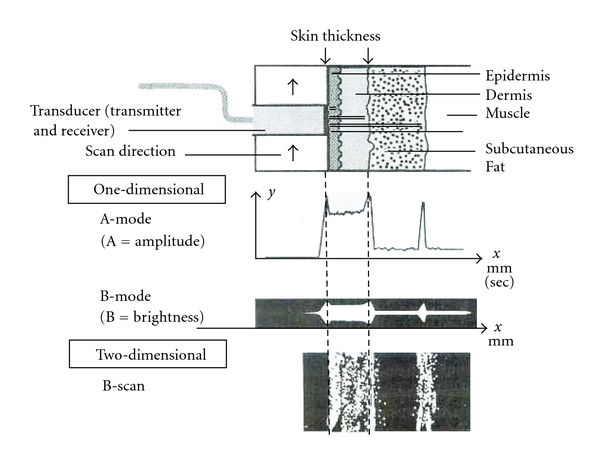
Schematic representation of steps used to produce two-dimensional sonographic cross-section of skin [42].

The main modes of scanning in commercial available trasducers are the A-scan and B-mode. In A-scan the amplitude scan maps the intensity of reflections encountered along a single line through the skin surface. The result is displayed in a graph that plots amplitude of each reflection by the reflection's depth and this mode can be used to assess skin thickness. In a B-mode the brightness display collects data from many individual A-scan soundings and presents it in a digitalized two-dimensional picture in which bright pixels represent strong reflections and dark pixels depict the absence of reflections (Figure 1).

With trasducers of 20 MHz the dermis appears echogenic with echoes originating from the fiber network comprised of collagen and elastic fibers; tissue atrophy and edema appear as echo-poor areas. Several skin diseases are characterized by changes both in the epidermis and in the dermis. Ultrasound scanning can be used in dermatological field to assess skin thickness, tissue edema, inflammatory conditions, and the extent of dermal and subcutaneous fibrosis as well as to monitor the course of wound healing [43–45].

In our work a 22 MHz ultrasound device DUB-system (tpm-Taberna pro medicum, Luneburg, DE), 72 *μ*m resolution, 7-8 mm penetration, was used; the B-mode and A-scan were used, respectively, to acquire skin images and to obtain measurements of skin tissues thickness.

### 2.3. 3*D* Imaging of the Skin

In this study, the *in vivo* Measurement of the Skin (PRIMOS^pico^) (GFMesstechnik GmbH, Teltow, Germany) was used to assess surface roughness of normal skin and striae.

**Figure 2:**
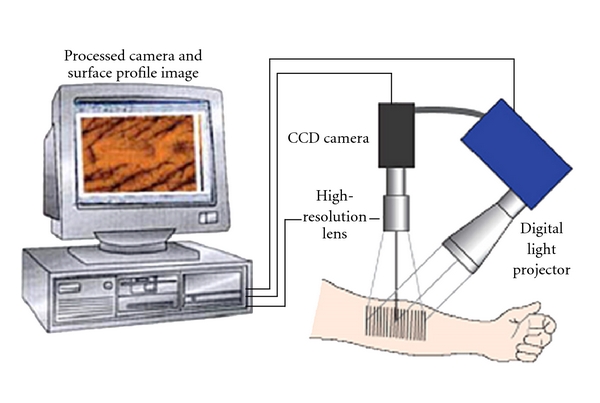
Principle of *in vivo* measurement of the skin.

Three-dimensional (3D) imaging is a recent measuring method in medicine. It uses optical projections, a high-resolution video camera, and computer software to rapidly generate images and measurements of skin topography. The method has been used successfully in wound care; dermatological laser treatments and its fundamental physics have been validated. The skin surface structure can be analyzed in the noncontact mode using the 3D optical system, as described in detail by Jacobi et al. [40]. This system is based on the digital stripe projection technique, which is used as an optical measurement process. A parallel stripe pattern is projected onto the skin surface and depicted on the CCD (charged-coupled device) chip of a camera through an optical system. The measurement system consists of a freely movable optical measurement head (with an integrated micromirror projector, a projection lens system, and a CCD recording camera), together with an evaluation computer (Figure 2). The 3D effect is achieved by the minute elevation differences on the skin surface, which deflect the parallel projection stripes. The measurements of these deflections represent qualitative and quantitative measurements of the skin profile.

We investigate both area treated with active and area treated with “placebo” at different times. The Primos system allowed us to evaluate, by matching, the same skin area in the three subsequent analysis times. After the measurements, 3-dimensional surface profiles were evaluated with the PRIMOS software. The 3D image acquired with the instrument can be transformed by the Primos 5.7 software to obtain a colored image in which each shade of color is related to a height (Figure 2).

The instrument also determines the roughness, which is based on the depth and the density of the skin furrows. Calculation of surface roughness was carried out within an area of 38.90 by 28.86 mm.

After filtering the photograph for hairs and the macrocurvature of body parts, the surface roughness was presented in numerous parameters. Preliminary research of these roughness parameters is maximum peak height (Rp) and maximum valley depth (Rv).

### 2.4. Statistical Analysis

*In order *to compare baseline values for skin thickness and skin roughness between the Active and Placebo areas, the Mann-Whitney nonparametric test was employed. One-way analysis of Variance (1 way ANOVA) test was used to compare skin values of all subjects at baseline, after 1 month of application, and at the end of the study. All statistical analyses were performed using GraphPad Prism software, version 5.0.

## 3. Results

### 3.1. Characterization Striae Type

**Figure 3:**
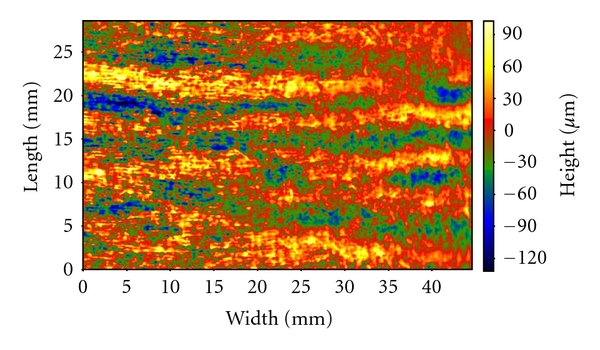
Striae rubra (yellow) and striae alba(green/blue).

The Primos Pico allowed us to obtain a colored images in which each shade of color is related to a height. In fact, the striae rubra appeared in yellow because it was in relief as for skin surface; on the contrary the striae alba appeared green/blue because it was depressed as for skin surface (Figure 3).

Thank to Primos analyses, all volunteers were then divided into three different groups in relation to stretch marks types. The 20% of the volunteers had a striae rubra, the 45% had a striae alba, and the 35% had both stria rubra then striae alba.

**Figure 4:**
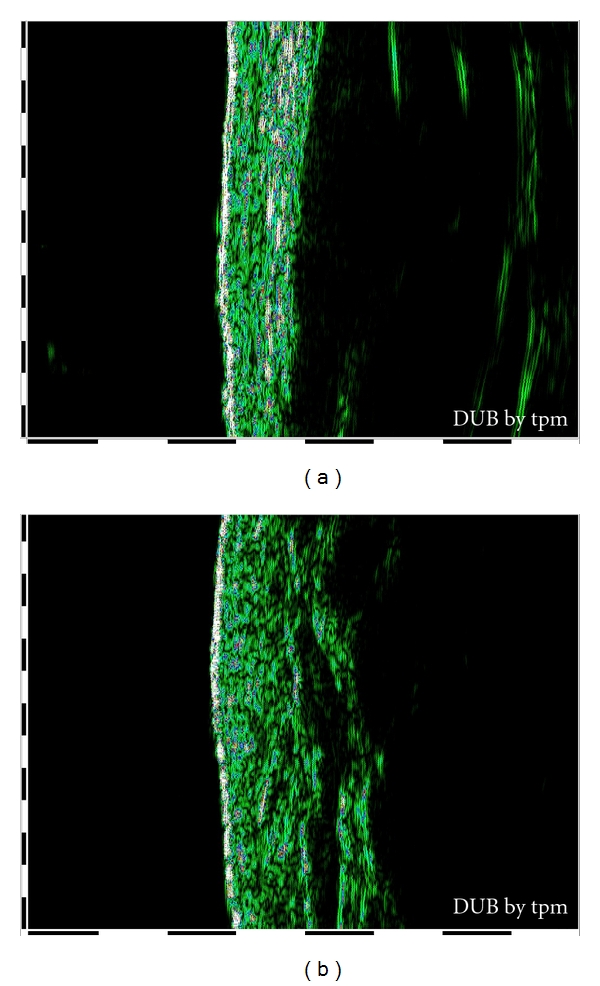
B-mode image of skin in which epidermis and dermis are visualized as echo-rich characteristics and striae as echo-low zone inside the dermis: (a) healthy skin; (b)skin with stretch marks.

By 22 MHz ultrasound imaging, it has been possible to visualize stretch mark as a poor echogenic zone localized into dermis, as shown in Figure 4.

Unlike the Primos, it has not been possible to distinguish the stretch marks type with the ulrasound imaging.

### 3.2. Evaluation of Topical Treatment Efficacy

#### 3.2.1. Ultrasound Scanning

**Figure 5:**
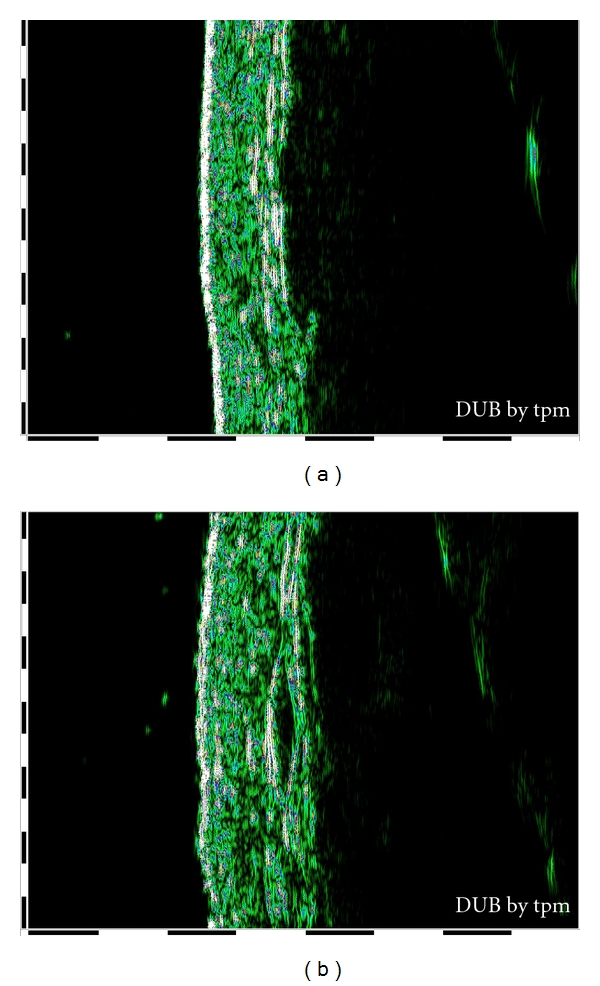
B-mode image of skin after two months of treatment: (a) skin treated with active product in which epidermis and dermis are visualized as echo-rich characteristics and a new white echo-rich zone appears instead of striae; (b) skin treated with placebo in which striae are visualized as echo-low zone.

Figure 5 shows images of a skin after 2 months of treatment with the active product Figure 5(a) and with the placebo Figure 5(b).

The measurements of skin thickness are performed using the A-scan mode on all subjects at baseline, after 1 month, and at the end of the study.

**Figure 6:**
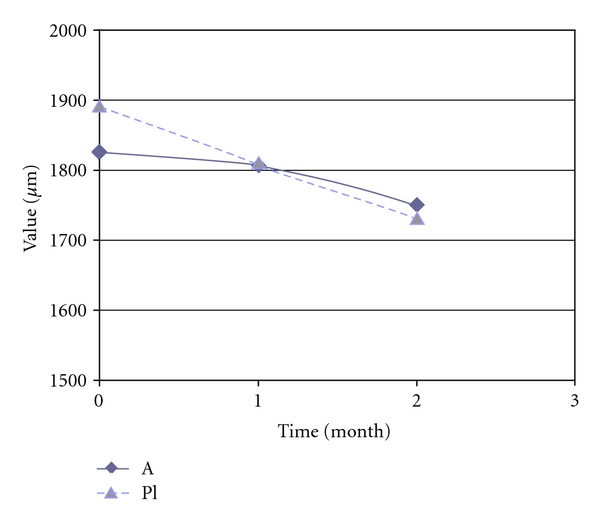
Curve trend of epidermis-dermis thickness in placebo and active-treated area.

Figure 6 shows a comparison graph of the epidermis-dermis thickness in zone treated with active, with respect to placebo area. Statistical analysis, obtained for both treatments, did not highlight a statistically significant reduction of the thickness. These results could be explained from the new white echo-rich zone appearing instead of striae that do not induce dermis thickness modification, at least after two months of treatment.

#### 3.2.2. Primos Results

Data for skin roughness to all assessment times were obtained by elaborations of 3D images. The data, including mean values and standard deviations, are summarized in Table 1.

Statistical analysis of skin roughness demonstrated that there was a statistically significant reduction of Rp parameter only in the active treated (A) group, and no statistically significant differences were measured on Rv parameter in both Pl (placebo) and A (active) group.

**Figure 7:**
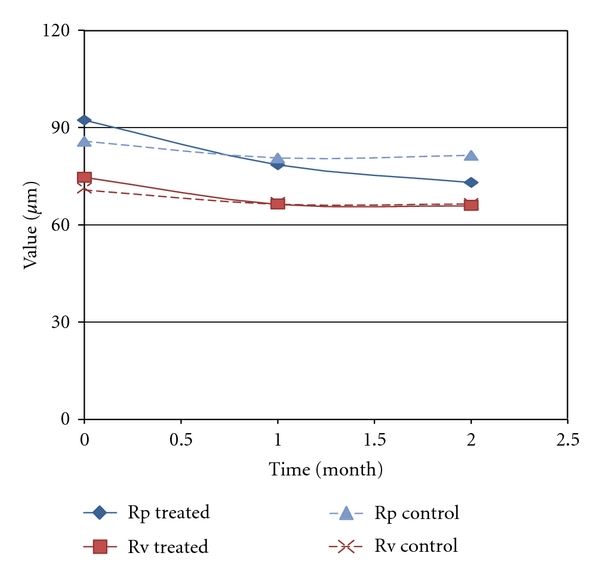
Curve trend of Rp and Rv in control and treated skin area.

In Figure 7 it is clear that, after two months of treatment, a statistically reduction of Rp value is obtained only in the active-treated groups.

For two volunteers the instrument was been used also to determine the total area, total volume, average depth of the SD of the skin. We selected volunteers with different types of striae in order to evaluate the effective of the treatment in immature and mature stretch marks. The results are summarized in Table 2.

**Figure 8:**
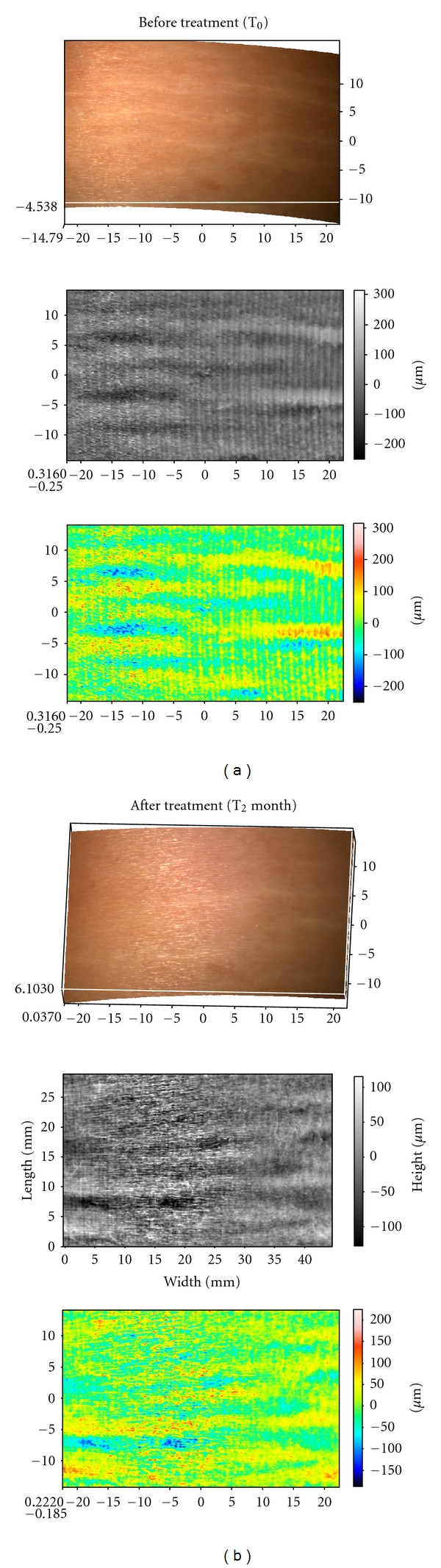
Stretch marks in 3D mode and in color scale and in grey scale images obtained before (a) and after two months of treatment (b).

The Primos system has allowed us to evaluate, by matching, the same skin area in the three subsequent analysis times. For example, Figures 8(a) and 8(b) show stretch marks in 3D mode and in color scale and in grey scale images, obtained at 0 and 2 months time. The images concern the area treated. Figure 8(a) shows a skin area selected before treatment: in the image in a color scale it is possible to visualize the striae rubra that appear as yellow length streaks, and this is because at the beginning of SD maturation the striae are in relief. In the same zone it is possible to note the striae alba in baby-blue/blue color that represent a depth region of the skin.

Figure 8(b) shows the same skin area represented after two months of treatment: in the image in a color scale is possible to visualize the modification of SD wave. After two months of treatment with active product the skin seems more homogeneous and the striae rubra appear less defined. The striae alba in baby-blue/blue color do not appear really modified at the end of the treatment.

## 4. Discussion

The present investigation was carried out on 20 healthy volunteers to monitor the striae distensae maturation and to evaluate their modification after treatment. Recent or immature SDs are flattened areas of skin with a pink-red hue that may be itchy and slightly raised. Stretch marks then tend to increase in length and acquire a darker purple color. Over time, they become white, flat, and depressed. Several treatments have been proposed; yet no consistent modality is available. Some authors have suggested that time is the only treatment for SD and that they return to normal over years, which is not true [2, 3]. The *in vivo* evaluation of human skin surface topography is of great interest for dermatological research because this type of topography represents the three-dimensional (3D) organization of the dermis and the subcutaneous tissue. The surface topography can be considered a mirror of the functional status of the skin. It is an expression of the possibility for the skin to respond to mechanical stimuli and threats. Various treatments exist to reverse or at least decrease the skin changes. In order to assess the success of a stretch marks treatment the depth, variation, and distribution of the furrows need to be measured prior and after application of the pharmaceutical or cosmetic product [40]. The PRIMOS is a reliable and valid objective tool for evaluating surface roughness in normal appearing skin, in burn scars, in facial acne scars, and in psoriasis lesion [39, 46, 47]. By means of the PRIMOS, striae distensae maturation and final outcome can be measured and documented.

By ultrasound imaging, it is possible to visualize and diagnose the striae that appear in a very different way with respect to other dermis diseases, such as adipose protusions of the subcutis.

The high-frequency ultrasound and the 3D image device, described in this work, can be successfully employed together in order to evaluate the efficacy of a topical treatment.

The results of this study highlighted that, after two months of treatment, the striae rubra seem to be less defined; so the skin appears more homogeneous. The striae alba in baby-blue/blue color do not appear really modified at the end of the treatment.

These results suggest, according to other authors, that effective treatment of SD could be instituted during the active stage; well before that the scarring process is complete (3, 22), that is, when SDs are identified like striae rubra. This was confirmed by statistical analysis of skin roughness that demonstrated how Rp value subjected a statistically significant reduction only in a treated group. In fact, the Rp value represents a maximum peak height in the area selected. Instead, Rv value, that represents maximum valley depth, doesnot subject variation. These results are in agreement with the foregoing results (Figure 7) and they could demonstrate that after two months, of treatment only the striae rubra can be treated successfully.

**Figure 9:**
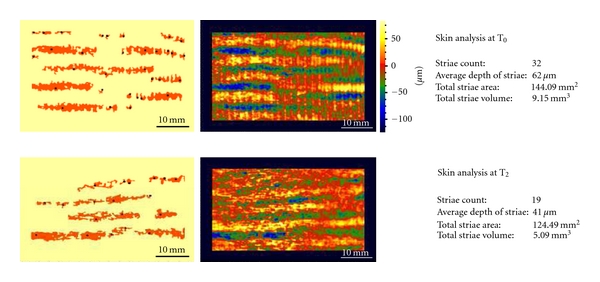
Skin analysis in a subject with striae rubra and striae alba before (*T*_0_) and after 2 months of treatment (*T*_2_).

**Figure 10:**
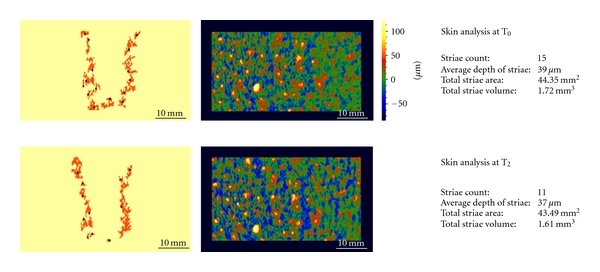
Skin analysis in a subject with striae alba before (*T*_0_) and after 2 months of treatment (*T*_2_). Again it could maintain that the treatment of striae alba didnot permit to obtain a reduction of this kind of stretch marks.

The instrument has been used also to determine the total area, total volume, and average depth of the SD in two volunteers in order to support the hypothesis aforesaid.  Figure 9 shows the results obtained at 0 and 2 months in a volunteer with striae rubra and alba; instead Figure 10 shows the results obtained at 0 and 2 months in a volunteer with striae alba.

## 5. Conclusions

Striae Distensae are a common skin condition that rarely causes some significant medical problems but are often a significant source of distress to those affected.

In summary, this work demonstrated that the PRIMOS device can measure and document striae distensae type and maturation.

Furthermore, the high-frequency ultrasound and the 3D image device, described in this work, can be successfully employed in order to evaluate the efficacy of a topical treatment.

In order to define guidelines for the application of these techniques in the stretch marks treatment, further studies enrolling more volunteers are necessary.

## Figures and Tables

**Table 1:** Mean values and standard deviation of skin roughness of striae treated with Active (A) or Placebo (Pl) product.

	Pl-*t*_0_ *μ*m (SD)	Pl-*t*_1_ *μ*m (SD)	Pl-*t*_2_ *μ*m (SD)	A-*t*_0_ *μ*m (SD)	A-*t*_1_ *μ*m (SD)	A-*t*_2_ *μ*m (SD)
Rp	85.8 (20.75)	80.63 (14.71)	81.45 (17.68)	92.3 (22.04)	78.5 (26.69)	73.05 (16.29)
Rv	71.35 (15.57)	66.94 (14.81)	67.05 (16.52)	74.8 (17.05)	66.45 (17.03)	66 (13.80)

**Table 2:** Value of total area, total volume, and average depth of the SD of the skin.

	Volunteer with striae rubra and alba		Volunteer with striae alba	
	*T*_0_	*T*_2_	*T*_0_	*T*_2_
Striae count	32	19	15	11
Average depth of striae	62 *μ*m	41 *μ*m	39 *μ*m	37 *μ*m
Total striae area	144.09 mm^2^	124.49 mm^2^	44.35 mm^2^	43.49 mm^2^
Total striae volume	9.15 mm^3^	5.09 mm^3^	1.72 mm^3^	1.61 mm^3^
